# Convenient syntheses of fullerynes for ‘clicking’ into fullerene polymers

**DOI:** 10.1080/15685551.2016.1256462

**Published:** 2016-11-11

**Authors:** Fu-Ai Teng, Yanli Guo, Jianping He, Yong Zhang, Zhewen Han, Hui Li

**Affiliations:** ^a^ School of Materials Science and Engineering, East China University of Science and Technology, Shanghai, P.R. China

**Keywords:** Fullerene polymer, Bingel reaction, ‘click’ chemistry, shape amphiphiles

## Abstract

Alkyne-functionalized fullerenes (fullerynes) were designed and conveniently synthesized via Bingel reaction in one step with high yields. They were used to react with azido-functionalized polystyrene (PS) via Huisgen [3 + 2] cycloaddition ‘click’ chemistry to form two fullerene polymers: one with C_60_ tethered to the end of a PS chain (C_60_-1PS) and the other with C_60_ tethered at the junction point of two PS chains of identical molecular weight (C_60_-2PS). The fullerene polymers were characterized by ^1^H NMR, ^13^C NMR, FT-IR, MALDI-TOF mass spectrometry and SEC. The results showed that the fullerene polymers are well-defined with narrow polydispersity and high fullerene functionality. Besides, aggregation of C_60_ in THF was observed in the SEC traces. The optical properties of the fullerene polymers were studied by UV–Vis absorption spectroscopy, and the results suggested that the PS chain(s) on the fullerene core has no remarkable effect on the optic property of C_60_. The thermal properties of the fullerene polymers were studied by TGA and DSC, and the results indicated that the two fullerene polymers with different C_60_ content and distinct molecular topology may have different self-assemble architectures in the solid state. The well-defined fullerene polymers can be used as model compounds to study the self-assemble architecture of shape amphiphiles based on polymer-tethered molecular nanoparticles.

## Introduction

Ever since its discovery and multi-gram availability, [60]fullerene(C_60_) has attracted intense research interest for its fascinating carbon nanostructures with unique photoelectronic and biological properties.[1–3] However, the poor compatibility of pristine fullerenes with other materials leads to its strong tendency toward aggregation and low solubility in common solvents and thus limits their facile derivatization and application.[1,4,5] To fully take advantage of its outstanding properties, it is critical to manipulate their interactions to form ordered structures in multi-dimensions across different length scales.[6] Attaching a small number of polymer tethers to the surface of C_60_ core provides a convenient way to do so.[7,8] Since C_60_ core are considered as a spherical molecular nanoparticles (MNP), the fullerene-polymer conjugates are model shape amphiphiles that have been predicted by computer simulation to exhibit rich phase behaviors and various unusual structures due to packing constraints and amphiphilic interactions.[8–10]

Recently, Cheng et al. has reported a robust way to synthesize well-defined fullerene polymers by ‘click’ chemistry.[5,11–13] The Cu-catalyzed Huisgen [3 + 2] cycloaddition reaction between the alkyne functionalized fullerene (thus named ‘fulleryne’) and azido-functionalized polymers proceeded efficiently at room temperature with no side reactions between azide and C_60_ core.[14–23] With the development of living polymerization technology, polymer with controlled molecular weight, narrow polydispersity can be easily synthesized.[24–28] However, the syntheses of fullerynes are usually nontrivial.[5] Several methods have been reported,[11,29–32] but most of them involves multi-step syntheses and drastic reaction conditions. In order for the ‘click’ chemistry to be viable for practical applications, it is important to find that fullerynes are readily available and highly reactive.[11] Bingel–Hirsch reaction has been considered as one of the most versatile and efficient methods for C_60_ functionalization under mild reaction conditions to afford stable methanofullerene products with high yields.[33–35] Nierengarten et al. [36–46] prepared C_60_ derivatives bearing terminal alkyne, and used them as building blocks in the efficient preparation of ‘sugar balls’ and other conjugates. By varying the number and positions of the alkyne groups on the surface of the C_60_ in fullerynes, fullerene materials with different architecture can be obtained from simple common precursors.[12,13,16]

**Scheme 1.**
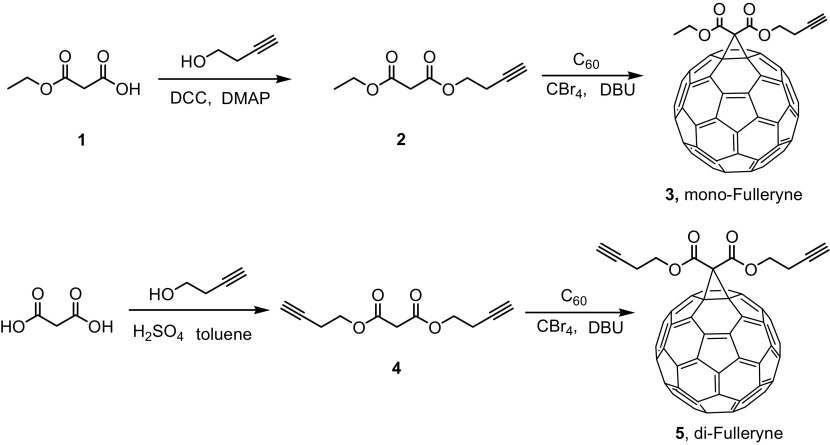
Preparation of mono-Fulleryne and di-Fulleryne.

**Scheme 2.**
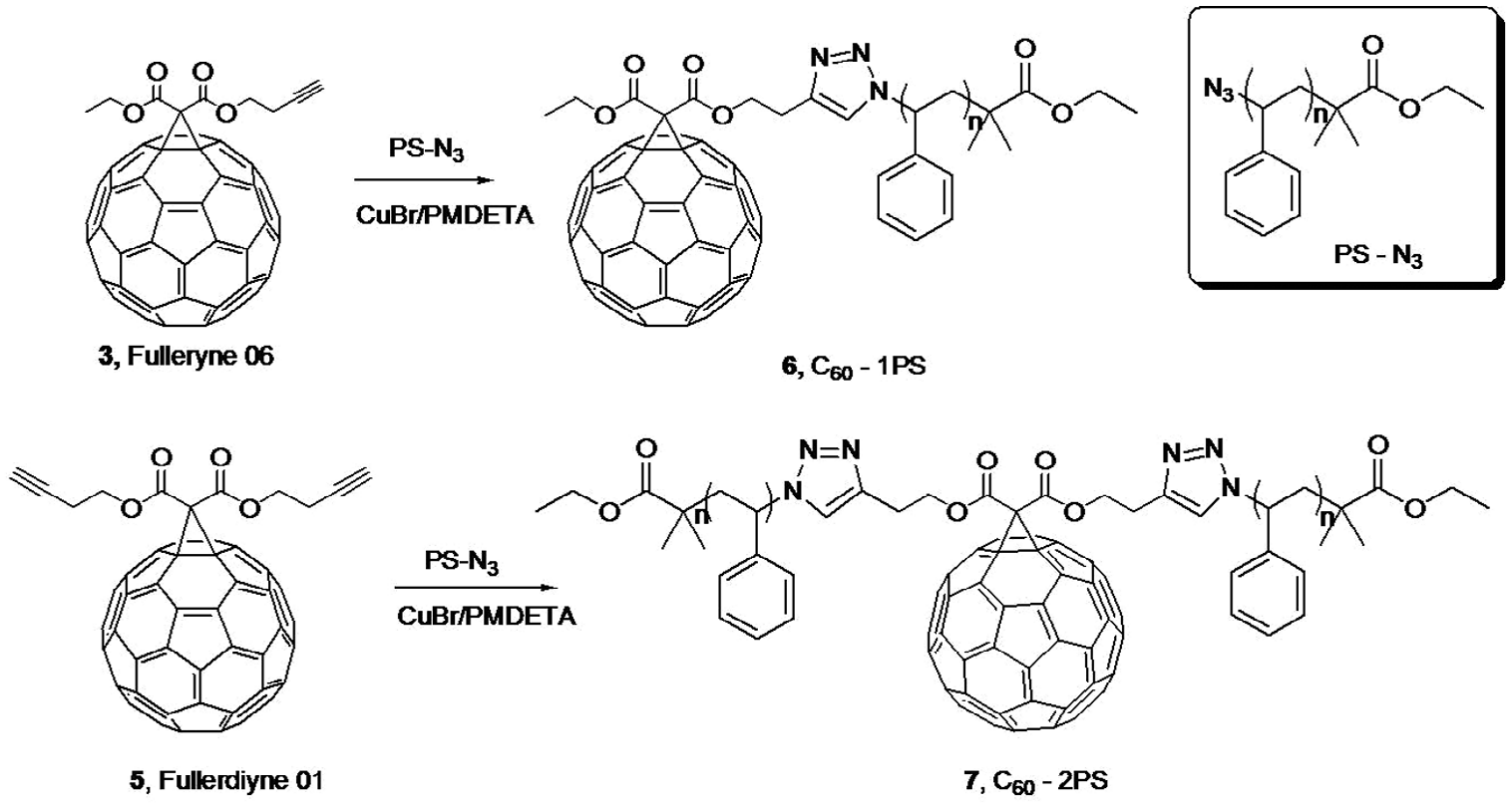
Synthesis of C_60_-PS and C_60_-2PS.The structure of PS-N_3_ is shown in box.

In this paper, we describe the use of Bingel reaction for the convenient syntheses of fullerynes in one step from C_60_ (Scheme 1) and the application of these fullerynes in the syntheses of two fullerene polymers of distinct topology (Scheme 2): one being C_60_ tethered with one polymer tail (C_60_-1PS) and the other being C_60_ tethered at the junction point between two identical polymer chains (C_60_-2PS). The fulleryne with one alkyne group is named mono-Fulleryne, and the fulleryne bearing two alkyne groups is named di-Fulleryne.

## Experimental procedure

### Materials and methods

C_60_ (MTR Ltd. >99.5%); malonic acid (SINOPHARM, ACS grade); 3-butyn-1-ol (J&K, 98%); diethyl malonate (SINOPHARM, ACS grade); carbon tetrabromide (CBr_4_, SINOPHARM, ACS grade); dicyclohexylcarbodiimide (DCC, J&K, 99%); dimethylaminopyridine (DMAP, J&K, 99%); 1,8-diazabicyclo[5.4.0]undec-7-ene (DBU, J&K, 98%); sodium azide (Alfa Aesar, 99%); *N,N,N′,N′,N″*-pentamethyldiethylenetriamine (PMDETA, Aldrich, >99%); petroleum ether (SINOPHARM, ACS grade); ethyl acetate (EtOAc, SINOPHARM, ACS grade); diethyl ether (SINOPHARM, ACS grade); methanol (SINOPHARM, ACS grade); acetone (SINOPHARM, ACS grade); cyclohexane (SINOPHARM, ACS grade); toluene (SINOPHARM, ACS grade); Tetrahydrofuran (THF, SINOPHARM, ACS grade) were used as received. Anhydrous dichloromethane (CH_2_Cl_2_, SINOPHARM, ACS grade), *N,N*′-dimethylformamide (DMF, SINOPHARM, ACS grade) were obtained by distillation from CaH_2_; anhydrous toluene were obtained by distillation from sodium. CuBr (SINOPHARM, ACS grade) was washed with 1 M sulfuric acid, anhydrous ethanol, anhydrous diethyl ether respectively before dried under vacuum. 3-Ethoxy-3-oxopropanoic acid (**1**), PS-N_3_ was prepared according to literature procedures.[5,47]

All ^1^H and ^13^C NMR spectra were acquired in CDCl_3_ (Aldrich, 99.8% D) using a Varian Mercury spectrometer operating at 400 and 100 MHz respectively at room temperature. The ^1^H NMR spectra were referenced to the residual proton impurities in the CDCl_3_ at *δ* 7.27 ppm and ^13^C NMR spectra were referenced to ^13^CDCl_3_ at *δ* 77.00 ppm.

Fourier transform infrared (FT-IR) spectra were taken using a Nicolet Magna-IR 550 FT-IR spectrometer in the range of 400–4000 cm^−1^ by making a KBr pellet from the mixture with the sample. The resolution was 4 cm^−1^ and 32 scans were averaged.

Matrix-assisted laser desorption/ionization time-of-flight (MALDI-TOF) mass spectra were recorded on a Kratos Axima CFR plus spectrometer (Shimadzu Biotech, Manchester, UK) with a 337 nm nitrogen laser and the acceleration voltage of 20 kv. The matrix was 2,5-dihydroxybenzoic acid (DHB). Mass spectra in positive ion mode were measured in both linear and reflection modes. Data analyses were conducted with Kompact software.

Size exclusion chromatography (SEC) analysis were performed using a Waters 515 Plus instrument equipped with three waters Styragel columns (103, 104, 105 Å) and three detectors (DAWN HELEOS, ViscoStar and Optilab rEX). THF was used as the eluent at a flow rate of 1.0 mL/min at 35 °C. The system was calibrated by a set of monodispersed standard polystyrenes.

UV–Vis spectra were measured on a CARY 100 UV–Vis spectrophotometer. Samples were prepared in THF at a concentration of 1 × 10^−5^ M, and the spectra were recorded between 200 and 600 nm.

Thermogravimetric analyses (TGA) were carried out with a NETZSCH STA 409 PC/PG thermogravimetric analyzer at a heating rate of 20 °C/min under a nitrogen atmosphere. Differential scanning calorimetry (DSC) was performed on DSC Q2000 V24.10 with a heating rate of 5 K min^−1^ under N_2_ flow.

### Typical synthetic procedures

#### Ethyl-3-butyn-1-yl malonate (**2**)

To a mixture of 3-ethoxy-3-oxopropanoic acid (**1**, 36.16 g, 0.274 mol), DMAP (16.74 g, 0.137 mol) and dry CH_2_Cl_2_ (100 mL), DCC (67.81 g, 0.329 mol) was added at 0 °C. At this temperature, the mixture was stirred for 30 min before 3-butyn-1-ol (23.04 g, 0.329 mol) was added. After stirring at 0 °C for another 2 h, the mixture was allowed to warm up to room temperature, and stirred overnight. The mixture was then filtered, and the filtrate was evaporated. The residues were dissolved in cool EtOAc (−16 °C) and filtered again. After solvent removal, the solids were further purified by column chromatography on silica gel with petroleum ether/EtOAc (10/1, v/v) to afford compound **2** as a yellow, viscous oil (17.98 g, 27.7%). ^1^H-NMR (CDCl_3_, 400 MHz,): *δ* ppm 4.17–4.29 (m, 4H), 3.40 (s, 2H), 2.56 (td, *J* = 6.8, 2.7 Hz, 2H), 2.02 (t, *J* = 2.7 Hz, 1H), 1.29 (t, *J* = 7.1 Hz, 3H). ^13^C-NMR (CDCl_3_, 100 MHz): *δ* ppm 166.28, 79.61, 70.11, 62.97, 61.52, 41.38, 18.76, 14.01. FT-IR (KBr) ν (cm^−1^): 3294.0 (≡C–H), 2986.0, 2929.7, 2855.3, 2117.2 (C≡C), 1725.3 (C=O). EI-MS: 184.1 ([M]^+^, calcd. for C_9_H_12_O_4_: 184.1).

#### Mono-Fulleryne (**3**)

A 500 mL three-necked flask equipped with dropping funnel was charged with C_60_ (360 mg, 0.50 mmol), CBr_4_ (199 mg, 0.86 mmol), and dry degassed toluene (200 mL). After C_60_ was dissolved, compound 2 (138 mg, 0.75 mmol) was added, and a solution of DBU (152 mg, 1.0 mmol) in toluene (40 mL) was then added dropwise during a period of 1 h. After stirring for another 6 h at room temperature under nitrogen in the absence of light, the mixture was concentrated and eluted with cyclohexane/toluene (3/2,v/v) on silica gel column. After the first fraction containing unreacted C_60_, mono-Fulleryne (**3)** was obtained as a dark solid (187 mg, 41.5%). ^1^H-NMR (CDCl_3_, 400 MHz,): *δ* ppm 4.53–4.64 (m, 4H), 2.77 (td, *J* = 6.7, 2.7 Hz, 2H), 2.06 (t, *J* = 2.7 Hz, 1H), 1.50 (t, *J* = 7.1 Hz, 3H). ^13^C-NMR (CDCl_3_, 100 MHz): *δ* ppm 163.69, 163.65, 145.54, 145.43, 145.30, 145.15, 144.94, 144.90, 144.13, 143.33, 143.22, 142.46, 142.18, 142.13, 141.20, 139.54, 139.11, 79.67, 71.69 (*sp* ^3^ C), 70.81, 64.98, 63.85, 52.28, 19.27, 14.54. FT-IR (KBr) ν (cm^−1^): 3298.1 (≡C–H), 2956.3, 2924.2, 2828.5, 2124.8 (C≡C), 1748.1 (C=O), 1233.6, 527.6 (C_60_, C–C). MALDI-TOF-MS: 902.9 ([M]^+^, calcd. for C_69_H_10_O_4_: 902.8).

#### Di(3-butyn-1-yl) malonate (**4**)

A 250 mL three-necked flask equipped with the dean-stark strap was charged with malonic acid (10.4 g, 0.10 mol), 3-butyn-1-ol (28 g, 0.40 mol), toluene (60 mL) and four drops of concentrated sulfuric acid as catalyst. The mixture was refluxed under nitrogen for 5 h before cooled to room temperature. Water and diethyl ether was added, and the organic layer was separated. The water layer was extracted with diethyl ether for three times, and then the combined organic layers were washed with brine. After dried over anhydrous Na_2_SO_4_, the solvent of the organic layer was removed under reduced pressure. The crude product was purified by column chromatography by using petroleum ether/EtOAc (5/1, v/v) as eluent to yield a colorless oil. (11.98 g, 57.7%). ^1^H-NMR (CDCl_3_, 400 MHz,): *δ* ppm 4.26 (td, *J* = 6.8, 1.3 Hz, 4H), 3.44 (s, 2H), 2.57 (dd, *J* = 8.9, 4.6 Hz, 4H), 2.04 (t, *J* = 2.7 Hz, 2H). ^13^C-NMR (CDCl_3_, 100 MHz): *δ* ppm 166.27, 79.90, 70.43, 63.30, 41.41, 18.99. FT-IR (KBr) ν (cm^−1^): 3294.9 (≡C–H), 2966.9, 2920.2, 2855.3, 2117.2 (C≡C), 1733.9 (C=O). EI-MS: 207.1 ([M-H]^+^, calcd. for C_11_H_12_O_4_: 208.1).

#### Di-Fulleryne **(5)**

The synthesis was performed similarly to that for **3** except using di(3-butyn-1-yl) malonate (**4**) instead of **2**. Yield:41.6%. ^1^H-NMR (CDCl_3_, 400 MHz): *δ* ppm 4.54 (t, *J* = 6.7 Hz, 4H), 2.71 (m, 4H), 1.99 (t, *J* = 2.6 Hz, 2H). ^13^C-NMR (CDCl_3_, 100 MHz): *δ* ppm 163.48, 145.54, 145.45, 145.39, 145.28, 145.18, 144.95, 144.91, 144.87, 144.14, 143.34, 143.28, 143.25, 142.45, 142.15, 141.22, 139.36, 79.67, 71.54 (*sp* ^3^ C) 70.83, 65.06, 51.92, 19.27. FT-IR (KBr) ν (cm^−1^): 3266.7 (≡C–H), 2957.0, 2924.2, 2849.2, 2117.0 (C≡C), 1748.1 (C=O), 1233.5, 524.8 (C_60_:C–C). MALDI-TOF-MS: 926.8 ([M]^+^, calcd. for C_71_H_10_O_4_: 926.9).

#### C_60_-1PS **(6)**

PS-N_3_ (*M*_n_ = 2900, 247.5 mg, 0.08 mmol), mono-Fulleryne (**3**, 45.1 mg, 0.05 mmol), CuBr (1 mg, 0.007 mmol) were combined in a 100 mL Schlenk flask before dry degassed toluene (50 mL) was added. The flask was subjected to three freeze-pump-thaw cycles and then two drop of PMDETA was added. The wine-red solution was stirred at room temperature for 2 days. The reaction mixture was concentrated, applied to the top of a short column of silica gel. The column was eluted with toluene/ethyl acetate (50/1, v/v) to give the product as a brown powder (161 mg, 80.6%). ^1^H-NMR (CDCl_3_, 400 MHz,): *δ* ppm 6.31–7.40 (m, 128H), 4.99–5.22 (m, 1H), 4.60–4.78 (m, 2H), 4.30–4.44 (m, 2H), 3.34–3.74 (m, 2H), 3.06–3.30 (m, 2H), 1.17–2.69 (m, 80H), 0.80–1.05 (m, 12H). ^13^C-NMR (CDCl_3_, 100 MHz): *δ* ppm 177.73, 163.72, 139.00–146.80 (C_60_ *sp* ^2^), 125.60–129.40, 71.77 (C_60_ *sp* ^3^), 66.08, 63.71, 60.19, 40.00–50.00, 32.21, 29.99, 25.81, 14.46. FT-IR (KBr) ν (cm^−1^): 3063.1, 3028.5, 2976.3, 2924.8, 2855.7, 1732.7, 1603.2, 1456.1, 1170.8, 1084.7, 1015.6, 755.9, 696.7, 531.7. SEC:PDI = 1.01. MALDI-TOF: 3768.2 ([26mer]^+^, calcd. C_60_-1PS_26_ for C_283_H_229_N_3_O_6_: 3768.0).

#### C_60_-2PS (**7**)

The synthesis was performed in a procedure similar to that for **6** except using **5** instead of **3.** Yield: 78.7%. ^1^H-NMR (CDCl_3_, 400 MHz,): *δ* ppm 6.18–7.44 (m, 268H), 4.85–5.15 (m, 2H), 4.42–4.61 (m, 4H), 3.33–3.72 (m, 4H), 2.93–3.15 (m, 4H), 1.15–2.62 (m, 168H), 0.79–1.06 (m, 18H). ^13^C-NMR (CDCl_3_, 100 MHz): *δ* ppm 177.73, 163.57, 139.00–142.00 (C_60_ *sp* ^2^), 125.60–129.40, 71.62 (C_60_ *sp* ^3^), 68.05, 66.07, 63.68, 60.24, 40.00–50.00, 29.99, 31.19, 25.00–27.00, 14.20. FT-IR (KBr) ν (cm^−1^): 3064.1, 3028.5, 2921.8, 2859.7, 1721.0, 1596.9, 1490.1, 1445.5, 751.9, 698.2, 527.8. SEC:PDI = 1.01. MALDI-TOF: 6866.7 ([27mer·H]^+^, calcd. C_60_-2PS_27_ for C_515_H_464_N_6_O_8_: 6865.4).

## Results and discussion

### Design and synthesis of mono-fulleryne and di-fulleryne

**Figure 1.**
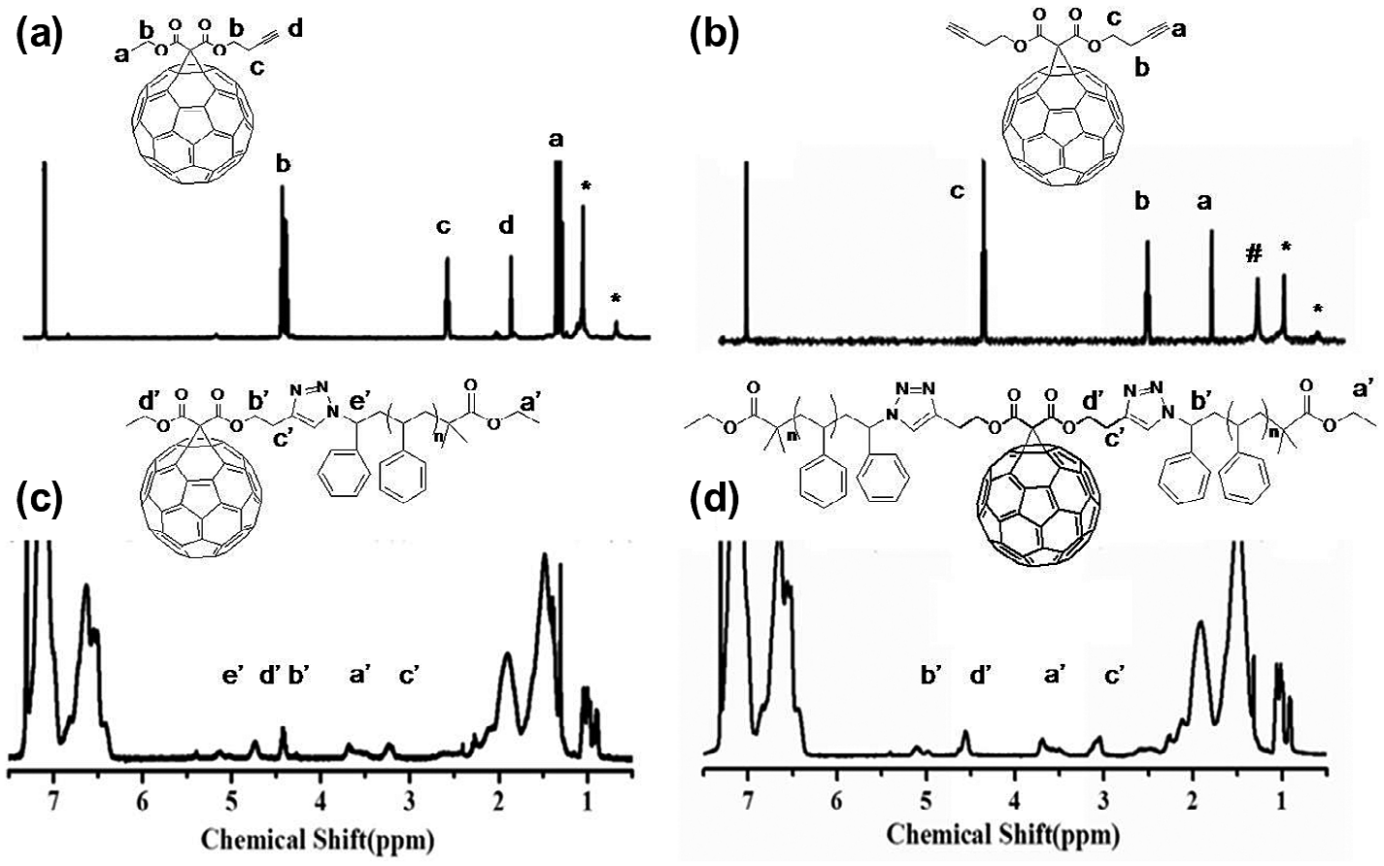
^1^H NMR spectra of mono-Fulleryne (a), di-Fulleryne (b), C_60_-1PS (c), and C_60_-2PS (d).

Note: The * and # denote the grease and water respectively.

**Figure 2.**
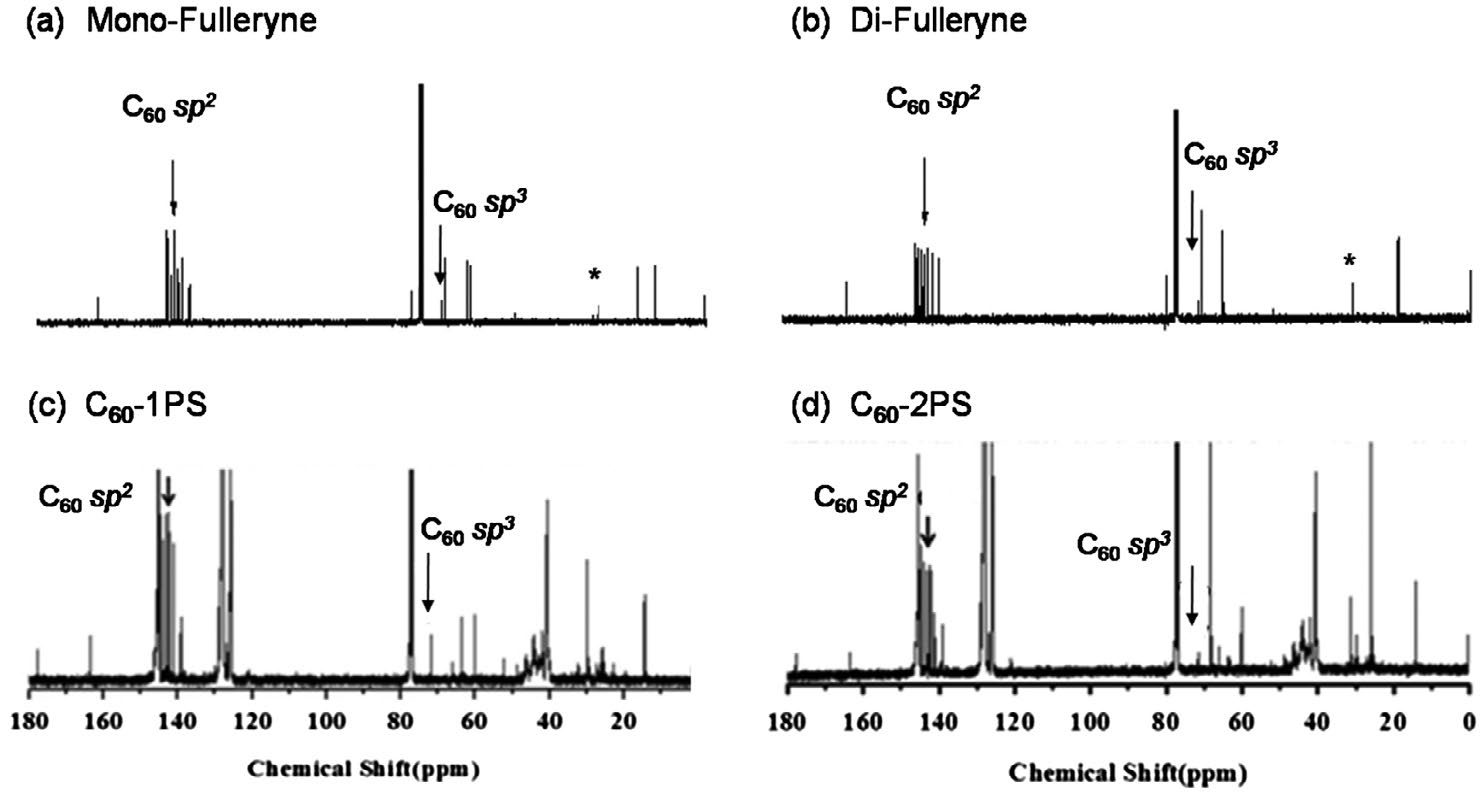
^13^C NMR spectra of mono-Fulleryne (a), di-Fulleryne (b), C_60_-1PS (c), and C_60_-2PS (d).

Note: The * denote the grease.

**Figure 3.**
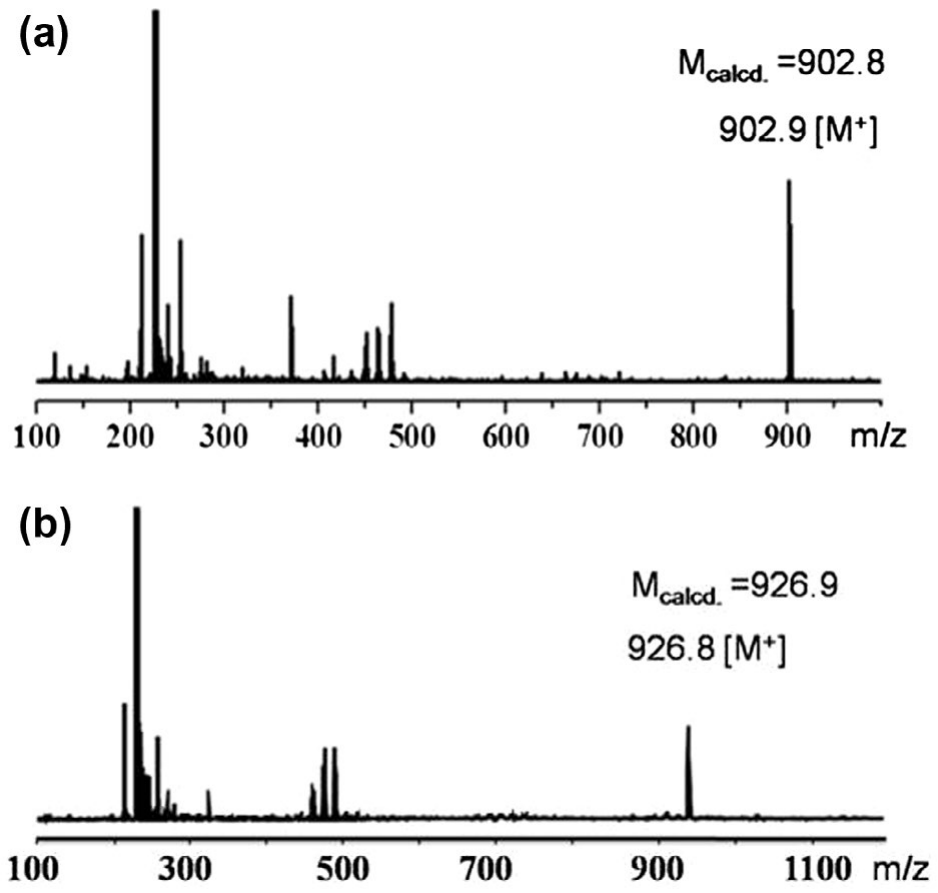
MALDI-TOF mass spectra of mono-Fulleryne (a), di-Fulleryne (b).

Fullerynes were synthesized in one step from C_60_ with high yield by reacting terminal-alkyne-functionalized malonates with fullerene via Bingel reaction (Scheme 1). The synthesis of mono-alkyne-functionalized malonate could be achieved by reacting alcohol with Meldrum’s acid as reported in literature [32,48] or the selective hydrolysis of diethylmalonate.[47] Acid **1** was synthesized as described in literature [47] and then reacted with 3-butyn-1-ol using Steglich esterification to afford ethyl-3-butyn-1-yl malonate (**2**) in a yield of 27.7%. Then, malonate **2** was directly reacted with fullerene in presence of CBr_4_ and DBU to give mono-Fulleryne (**3**) as a brown powder. The yield of 41.5% is good for fullerene functionalization. The synthesis of both malonate **2** and mono-Fulleryne were conveniently performed under mild reaction conditions. In literature, bifunctional fulleryne has been reported by Nierengarten and was synthesized via Bingel reaction. It was used as building blocks for copper-catalizedazide-alkyne cycloaddition (CuAAC) reaction.[37] Here we report a similar structure, di-Fulleryne. Bifunctional malonate (**4**) was synthesized by Fisher esterification of malonic acid with 3-butyn-1-ol in a yield of 57.7%. Its reaction with C_60_ under Bingel–Hirsch condition gives a bifunctional fulleryne, the di-Fulleryne. The yields for mono-Fulleryne and di-Fulleryne are very close. Both fullerynes were fully characterized by ^1^H-NMR, ^13^C NMR, FT-IR and MALDI-TOF mass spectrometry. In the ^1^H-NMR spectra (Figure 1(a) and (b)), resonance signals attributed to the α-protons of the malonate (~3.5 ppm) disappeared completely, evidencing the success of the Bingel reaction. Signals attributed to the *sp* ^*2*^ and *sp* ^3^ carbons of C_60_ can be clearly observed in the ^13^C NMR spectra (Figure 2(a) and (b)). The characteristic absorbance for C–C vibration on C_60_ can be clearly observed at 525 cm^−1^ in the FT-IR spectra. The MALDI-TOF mass spectra (Figure 3) further confirm the structures, where the observed molecular ion peaks have *m/z* values matching that of the calculated monoisotopic masses. All of the evidence clearly proves the chemical structure and purity of the new fullerynes.

### ‘Clicking’ for fullerene polymers

Azido-functionalized polystyrene can be facilely synthesized by atom transfer radical polymerization (ATRP) and subsequent nucleophilic substitution as reported.[5] ‘Click’ chemistry was then performed at room temperature with CuBr/PMDETA as the catalyst by reacting fullerynes with azido-functionalized polystyrene (Scheme 2). Recently, Yu et al. reported the synthesis of fullerene polystyrene shape amphiphiles using hydrophilic fullerene as the polar head and polystyrene as hydrophobic tail.[12] Versatile self-assembled micellar morphologies were observed and can be tuned by changing various parameters, such as molecular topology, polymer tail length and the molecular concentration. The C_60_ was used mainly as a structural scaffold in this work and the electronic properties of C_60_ were largely lost in these shape amphiphiles. It is of great interest to study the self-assemble behavior of shape amphiphiles containing pristine C_60_s. Thus, we try to synthesize well-defined fullerene polymers with distinct topologies as model shape amphiphiles.

The fullerene polymers synthesized using mono-Fulleryne and di-Fulleryne were also found to be a brown power that is readily soluble in common organic solvents such as THF, CH_2_Cl_2_ etc. The number and position of the tethered polymer tails are pre-determined by the location and number of alkyne groups on fullerynes, leading to shape amphiphiles of distinct architectures: C_60_-1PS and C_60_-2PS.

**Figure 4.**
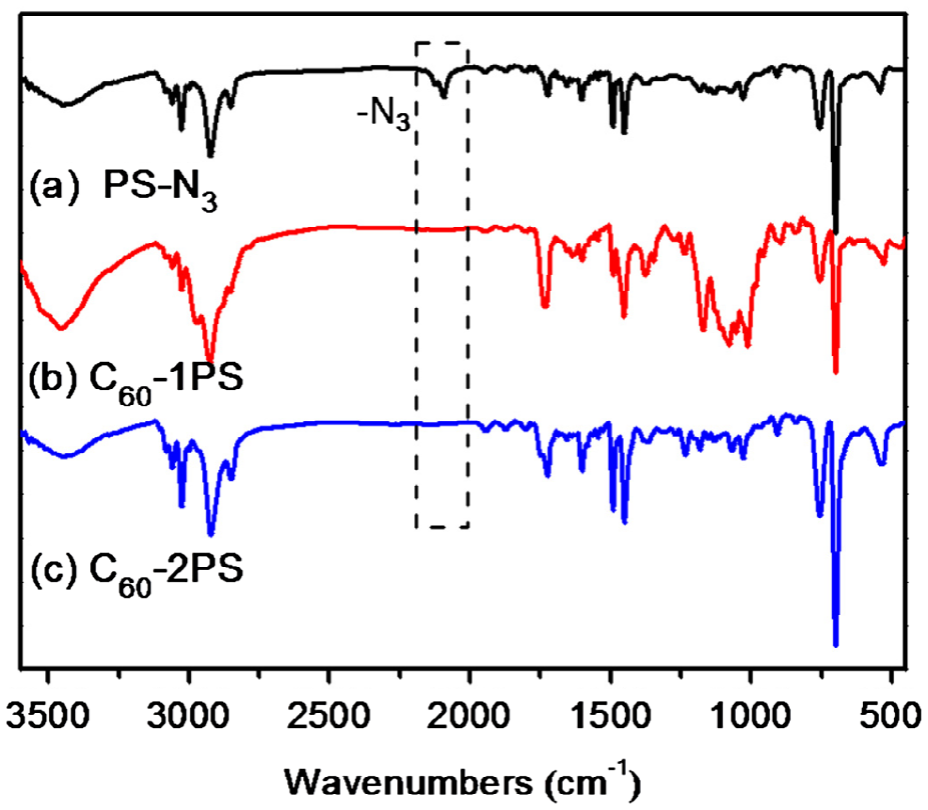
FT-IR spectra of PS-N_3_, C_60_-1PS, and C_60_-2PS.

**Figure 5.**
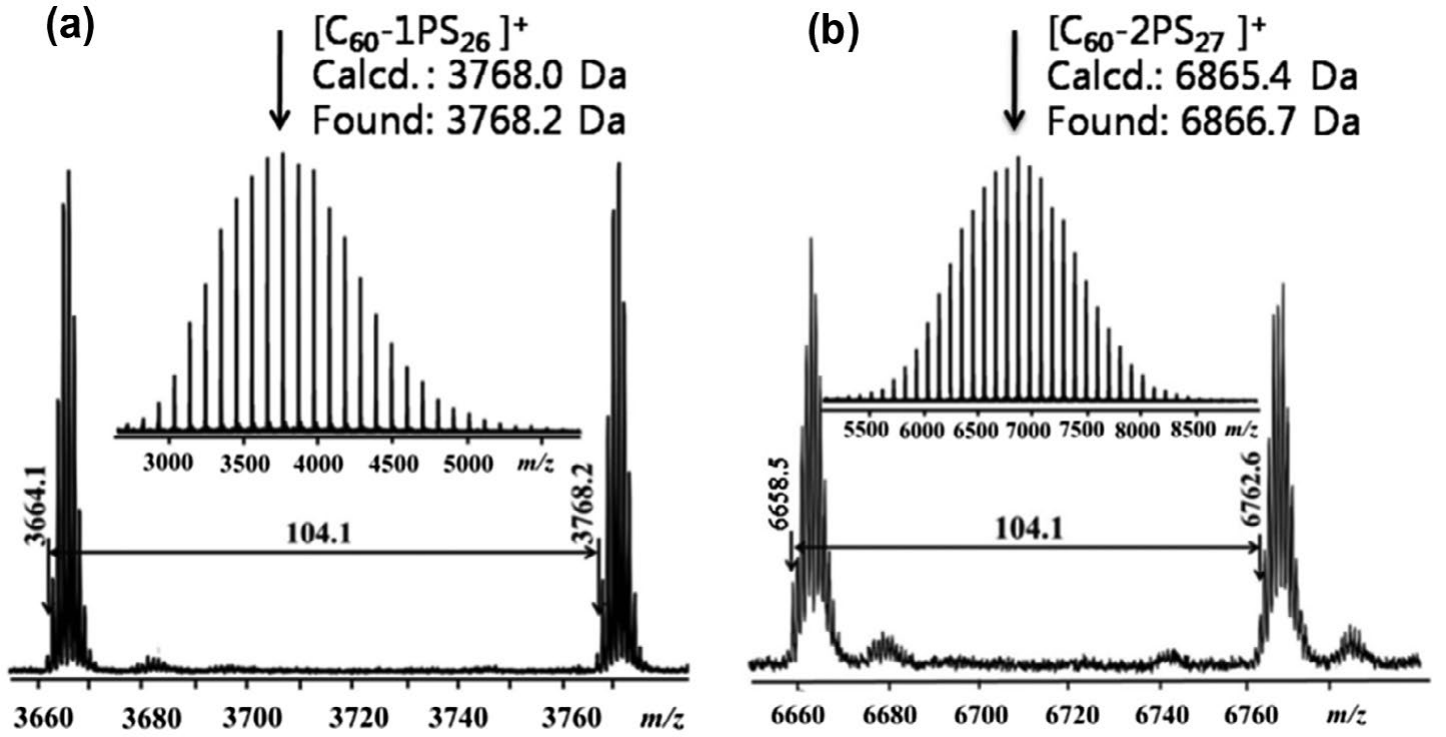
MALDI-TOF mass spectra for C_60_-1PS (a), and C_60_-2PS (b).

The fullerene polymers were fully characterized by ^1^H NMR, ^13^C NMR, FT-IR and MALDI-TOF mass spectrometry. The product exhibited the characteristic resonances of protons both near the 1,2,3-triazole (4.9–5.2, 3.0–3.3 ppm) and C_60_ unit (4.3–4.8 ppm) in the ^1^H-NMR spectra (Figure 1(c) and (d)). However, the signal attributed to the proton on the triazole ring cannot be observed, which may be overlapped with the peaks of the aromatic protons of PS.[36,49] Nevertheless, the success of the ‘click’ reaction and the precisely defined structures of both C_60_-1PS and C_60_-2PS can be validated from the combination of other techniques. In the ^13^C NMR spectra (Figure 2(c) and (d)), the signals attributed to the alkyne carbons (79.8 and 70.8 ppm) on fullerynes disappeared completely, and the *sp* ^3^ carbons as well as *sp* ^2^ carbons of the C_60_ moiety can be observed. FT-IR spectra (Figure 4) showed the complete disappearance of the azide group (2095.2 cm^−1^) and the appearance of a sharp peak at 525 cm^−1^, which is the characteristic for C_60_.[1] The MALDI-TOF mass spectra (Figure 5) showed a single Gaussian distribution with molecular weight in accordance to the proposed structure, confirming the stability and purity of the resulting fullerene polymers. The Gaussian distribution of the molecular weights was due to the different chain lengths of the polymers obtained during the polymerization. The difference in molecular weight between the two neighboring peaks was 104.1, which equals exactly to that of a single styrene repeating unit. The PDI calculated from MALDI-TOF is 1.01 for both C_60_-1PS and C_60_-2PS, which is very narrow. The results indicate that the fullerene polymers are pure with high C_60_ functionalization and well-defined as designed.

**Figure 6.**
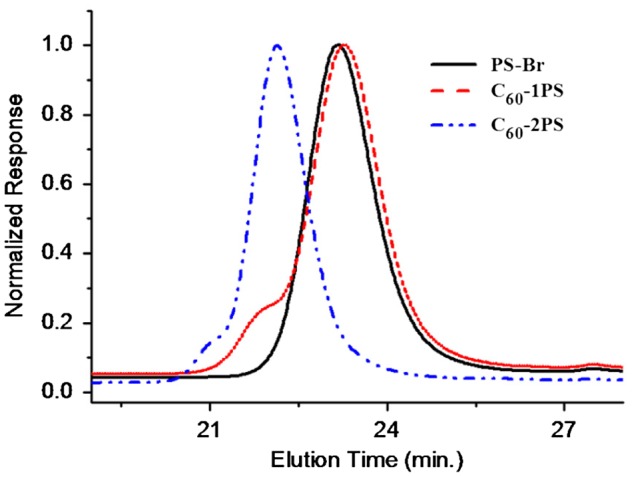
SEC traces of PS-Br, C_60_-1PS, and C_60_-2PS.

In the SEC traces (Figure 6), the retention time of C_60_-1PS was slightly larger than that of PS–Br. Zhang et al. explained that the polystyrene chain tends to wrap around the fullerene ball due to the insoluble of C_60_ in THF, thus decreasing the hydrodynamic volume of the fullerene polymer.[5] The C_60_-2PS showed a smaller retention volume, due to its larger molecular weight. A high molecular weight shoulder peak was observed in both fullerene polymers. The shoulder may be caused by the aggregation of C_60_ in THF,[11,13,50] as the fullerene polymers were well defined without multi-addition product evidenced form the high sensitive MALDI-TOF. The shoulder peak of C_60_-2PS is smaller than that of C_60_-1PS indicating the tendency of aggregation in C_60_-2PS is weaker. Interestingly, previous study by Zhang et al. did not show the aggregation phenomena of the PS-C_60_ polymer.[5] So we predicted that the aggregation of our C_60_-PS polymers may caused by the high concentration of the fullerene polymer solution or the low dissolve time in the SEC test, which need a systemic study in the further work. Ignoring the shoulder peak, the PDI calculated from SEC is 1.01 for both C_60_-1PS and C_60_-2PS, evidencing the well defined of the fullerene polymers.

**Figure 7.**
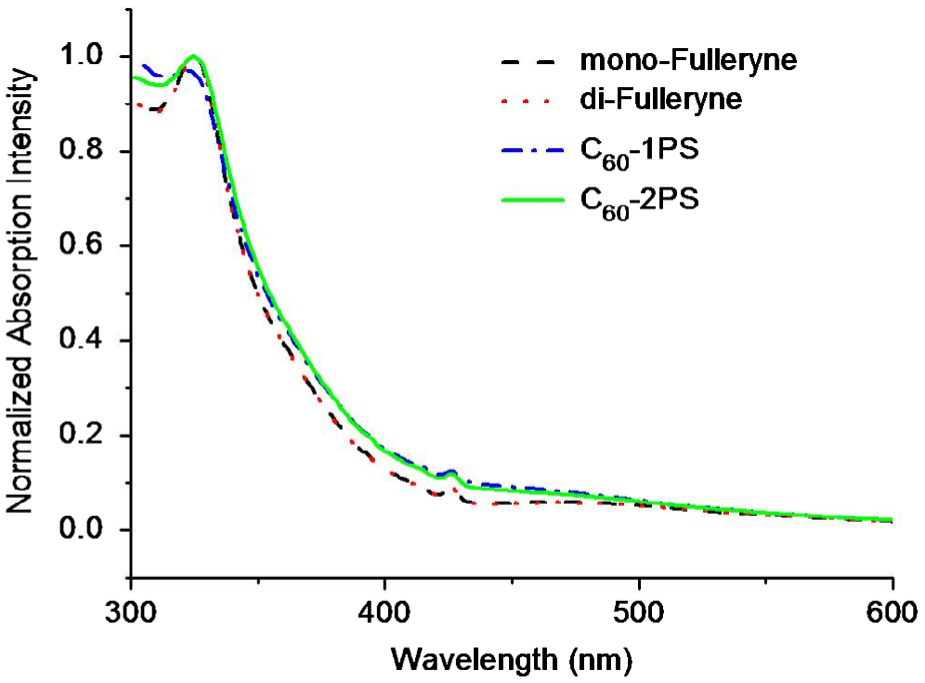
The steady-state UV–Vis absorption spectra of Mono-Fulleryne, Di-Fulleryne, C_60_-1PS and C_60_-2PS, measured in THF.

The steady state UV–Vis absorption spectra were measured in THF at room temperature. Figure 7 displays the absorption spectra of the alkyne-functionalized fullerenes, and the fullerene polymers. In the UV–Vis spectra of fullerynes, the absorption peaks at ~330 and 430 nm were typical fingerprints of C_60_ monoadducts (methanofullerene).[51–53] The UV–Vis absorption spectra of both C_60_-1PS and C_60_-2PS are very similar with that of fullerynes, indicated that the PS chain has no remarkable effect on the optic property of C_60_.

**Figure 8.**
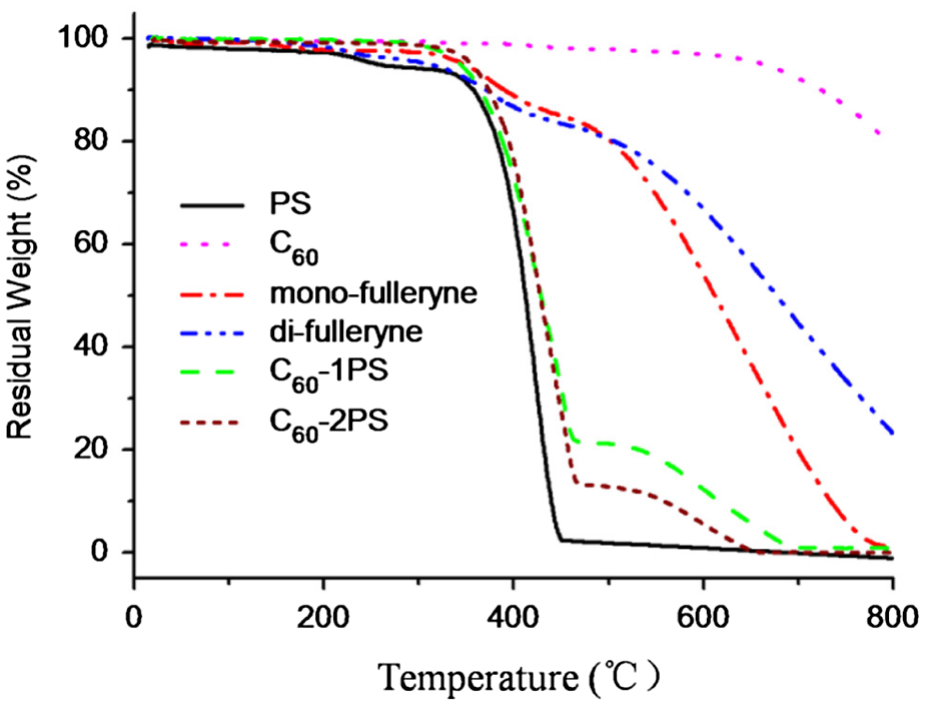
TGA thermograms of PS, C_60_, mono-fulleryne, di-fulleryne, C_60_-1PS and C_60_-2PS, under nitrogen atmosphere.

**Figure 9.**
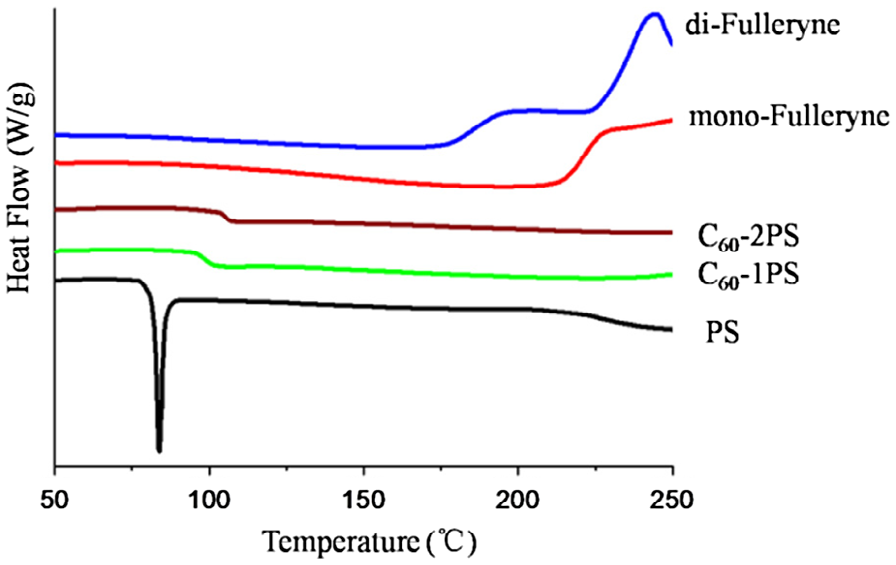
DSC thermograms of PS, mono-fulleryne, di-fulleryne, C_60_-1PS and C_60_-2PS.

The thermal properties of the alkyne-functionalized fullerenes and the fullerene polymers were studied by TGA and DSC (Figures 8 and 9). C_60_ and polystyrene were used as controls. The pristine fullerene exhibits outstanding thermal stability when heated to 550 °C. In comparison, the residue of mono-fulleryne and di-fulleryen at this temperature is 80.9 and 79.7% respectively, which is in good agreement with the theoretical estimation if assuming that the ‘non fullerene’ moiety had been completely decomposed and removed (79.7% for mono-fulleryen and 77.7% for di-fulleryne). The thermal decomposition of the alkyne-functionalized fullerene occurred at 330 and 328 °C respectively. Before this temperature, mono-fulleryne showed two distinctive exotherms process between the region 150–300 °C in the DSC profile, which may be attributed to the cross-linking reaction of the alkyne group. Di-fulleryne showed a similar DSC profile (thermal behavior) as that of mono-fulleryne, except that the exothermal peaks appeared at a lower temperature, indicating the lower thermal stability of di-fulleryene. Polystyrene undergoes a thermal decomposition between 386 and 450 °C. The onset decomposition temperature of the fullerene polymers (374 °C for C_60_-1PS and 382 °C for C_60_-2PS) are very similar with that of PS, indicated that the existence of C_60_ has no remarkable effect on the thermal stability of the PS chain. The residue of the fullerene polymer at 550 °C (18.9% for C_60_-1PS and 10.7% for C_60_-2PS) can be considered as the fullerene part, which is in good agreement with the theoretical content of fullerene in the polymer (19.1% for C_60_-1PS and 10.5% for C_60_-2PS). In the DSC profile, PS showed a melting point at 84 °C. In contrast, only one glass transition process was detected for fullerene polymers, indicated that the incorporation of C_60_ restricts the crystal of the PS chain. The *T*_g_ of fullerene polymers was reported to be varied with C_60_ content.[24,54–57] In our case, the *T*_g_ of C_60_-1PS (99 °C) is lower than the *T*_g_ of C_60_-2PS (105 °C), whereas the C_60_ content in C_60_-1PS is higher. This indicated that the two fullerene polymers with different C_60_ content and distinct molecular topology may have different self-assemble architectures in the solid state.

## Conclusions

In summary, mono-Fulleryne and di-Fulleryne were designed and conveniently synthesized in one step via Bingel reaction. These fullerynes were employed for ‘click’ into two fullerene polymers of distinct topology: one being C_60_ tethered with one polymer tail (C_60_-1PS) and the other being C_60_ tethered at the junction point between two polymer chains (C_60_-2PS). The fullerene polymers were well defined with narrow polydispersity and high degree of C_60_ functionalization. The PS chain(s) on the fullerene core has no remarkable effect on the optic property of C_60_. Aggregation of C_60_ in THF was observed in the SEC traces. The thermal behavior studies indicated that the two fullerene polymers with different C_60_ content and distinct molecular topology may have different self-assemble architectures in the solid state. The well-defined fullerene polymers can be used as model compounds to study the self-assemble architecture of shape amphiphiles based on polymer-tethered molecular nanoparticles.

## Disclosure statement

No potential conflict of interest was reported by the authors.

## Funding

This work was supported by the International Science & Technology Cooperation Program of China [grant number 2010DFB70470].
